# Supplementary material for: Antioxidant Efficacy and “In Vivo” Safety of a Bentonite/Vitamin C Hybrid
Source: Pharmaceutics. 2023 Apr 7;15(4):1171. doi: 10.3390/pharmaceutics15041171 (PMC10144955; doi:10.3390/pharmaceutics15041171)

**Supplementary Materials**

Antioxidant efficacy and “in vivo” safety of a bentonite/vitamin C hybrid

Dayaris Hernández, Anaela Montalvo, Irela Pérez, Clarence Charnay, Rita Sánchez-Espejo, Pilar Cerezo, César Viseras, Serena Riela, Giuseppe Cinà, Aramis Rivera

(S1) LAA/smectites systems: critical revision

In two of the references cited near the end of the introduction of the manuscript [1,2], the authors basically discuss the use of natural montmorillonite (Mt) and acid treated Mt in the intercalation and stabilization of ascorbic acid (LAA) for potential uses (not defined). In the first study, despite that a new method is declared in the last part of the introduction, is not properly described later in the article. The data corresponding to LAA load on the clay is not reported either. The second article focuses on the stabilization of the activity of LAA in dissolution at different natural and modified Mt concentrations. The third reference cited in our manuscript [3], studies the employment of natural and cation modified smectites as supports for pollutants removal, where the LAA was chosen as model emergent contaminant. Although in this investigation different parameters ―pH, LAA initial concentration from and time― were optimized, the following elements were not considered: 1) No details are given on the type of smectite evaluated, 2) At the optimal values of pH = 5 and pH = 8, the LAA species (pka = 4.2) are negatively charged. Additionally, at basic pH the LAA degradation is favored damaging its stability and antioxidant capacity.

References

1. Chen, B.-Y.; Lee, Y.-H.; Lin, W.-C.; Lin, F.-H.; Lin, K.-F. Understanding the characteristics of L-ascorbic acid-montmorillonite nanocomposite: Chemical structure and biotoxicity. *Biomed. Eng-App. Bas. C.* **2006**, *18*.

2. Lee, Y.-H.; Chen , B.-Y.; Lin, K.-Y.; Lin, K.-F.; Lin, F.-H. Feasibility study of using montmorillonite for stability enhancement of L-ascorbic acid. *J. Chin. Inst. Chem. Eng.* **2008**, *39*, 219-226.

3. Anouar, F.; Elmchaouri, A.; Taoufik, N.l.; Rakhila, Y. Investigation of the ion exchange effect on surface properties and porous structure of clay: Application of ascorbic acid adsorption. *J. Environ. Chem. Eng.* **2019**, *7*, 103404.

(S2) Ophthalmic irritability


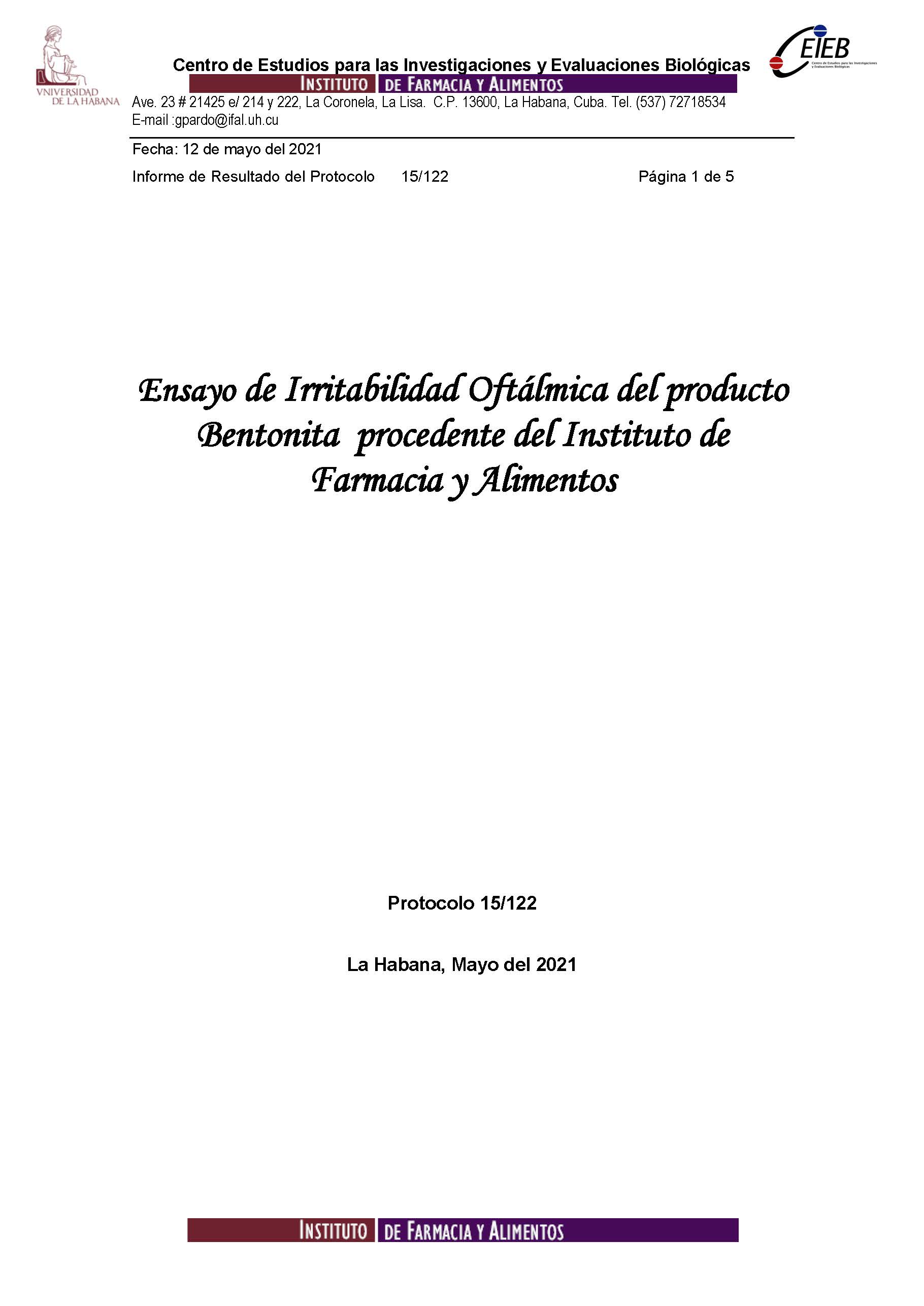


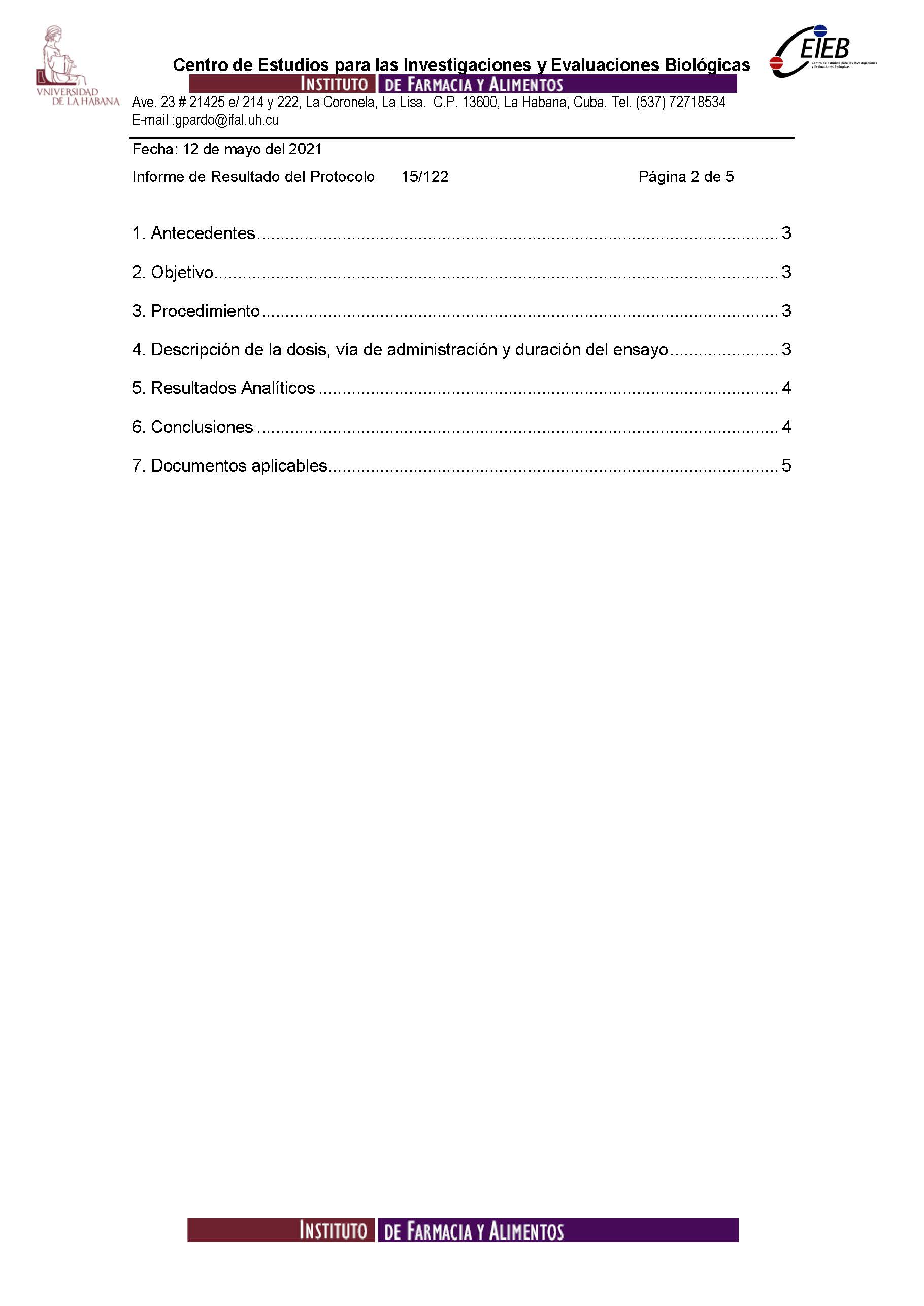

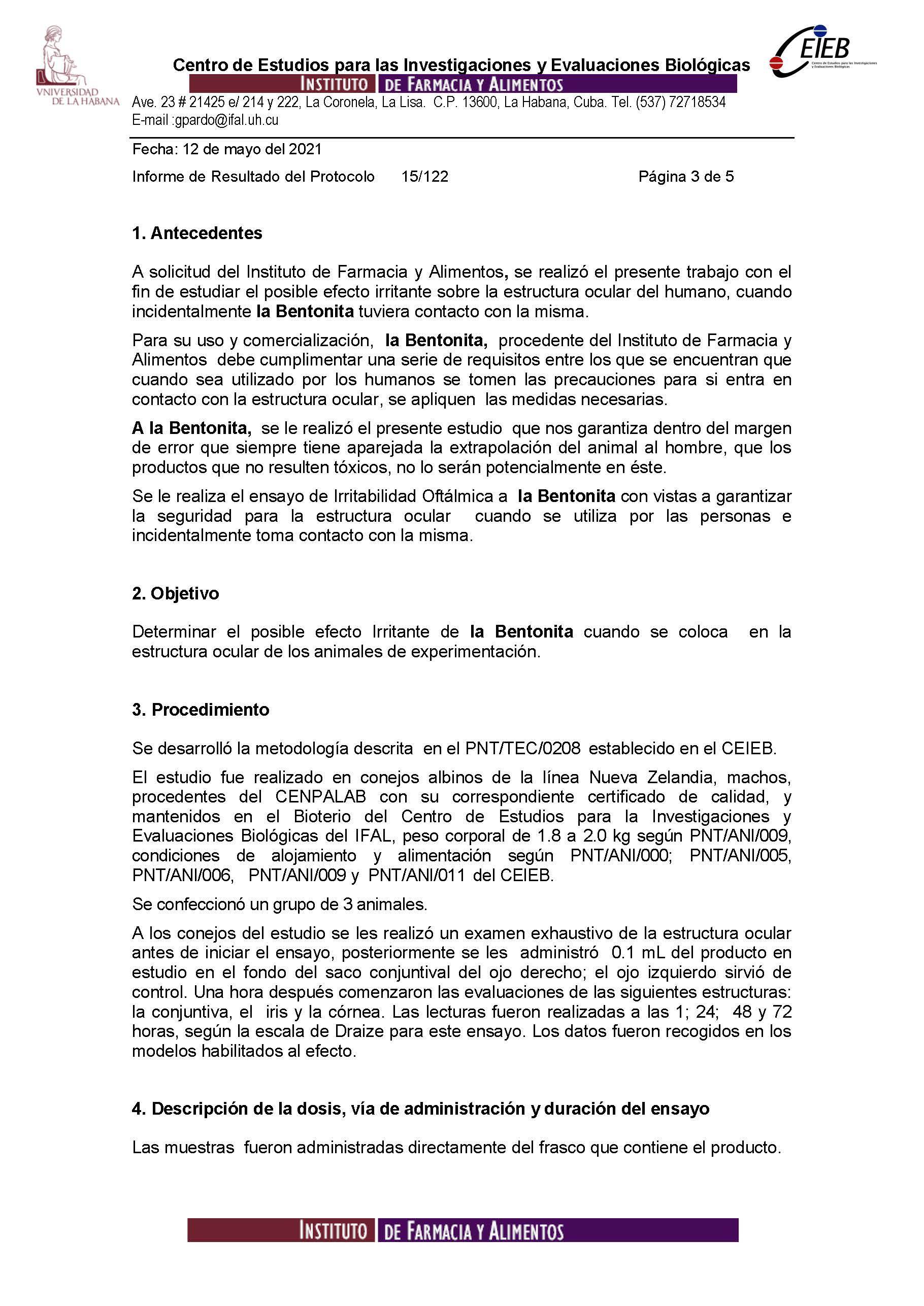

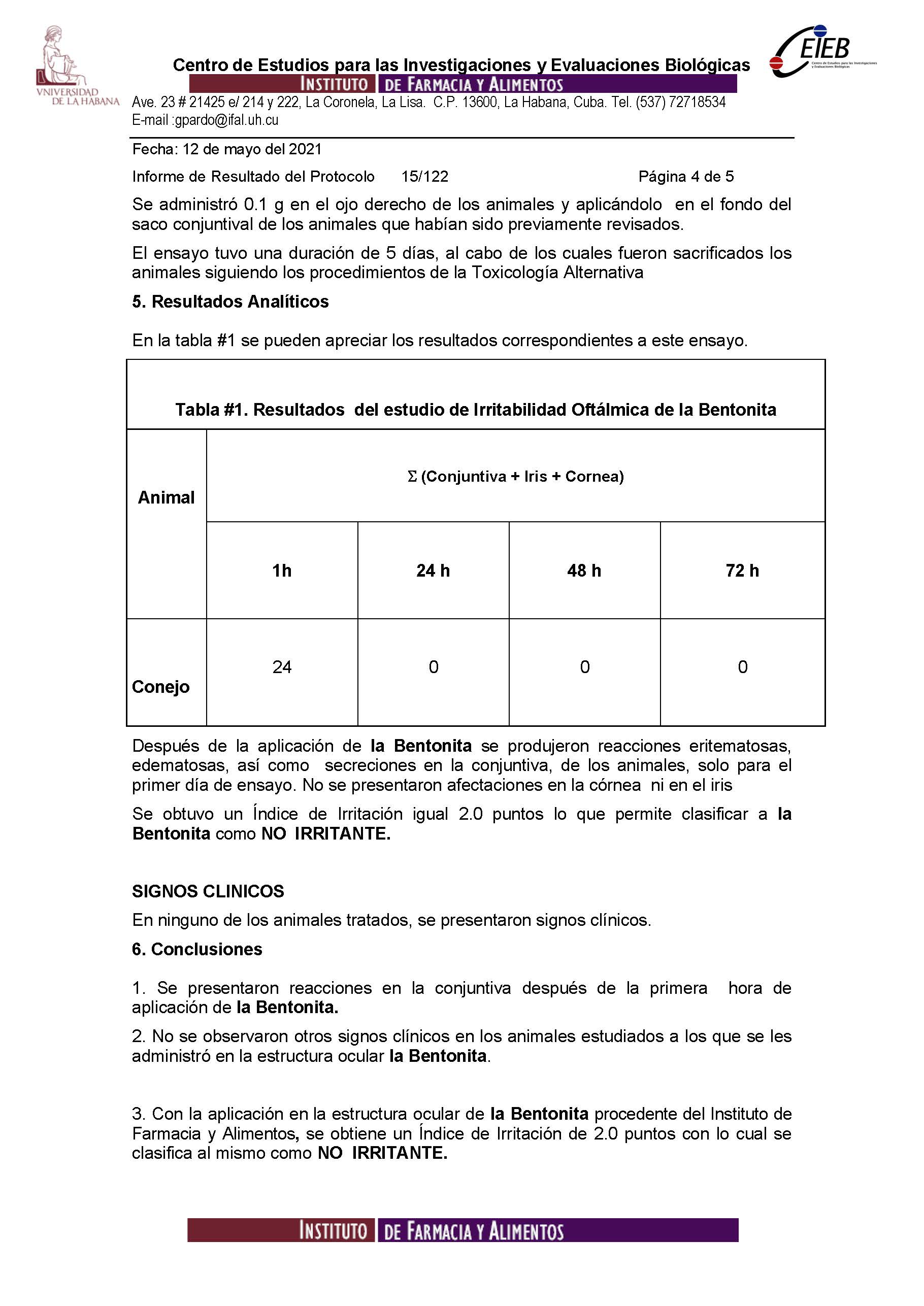

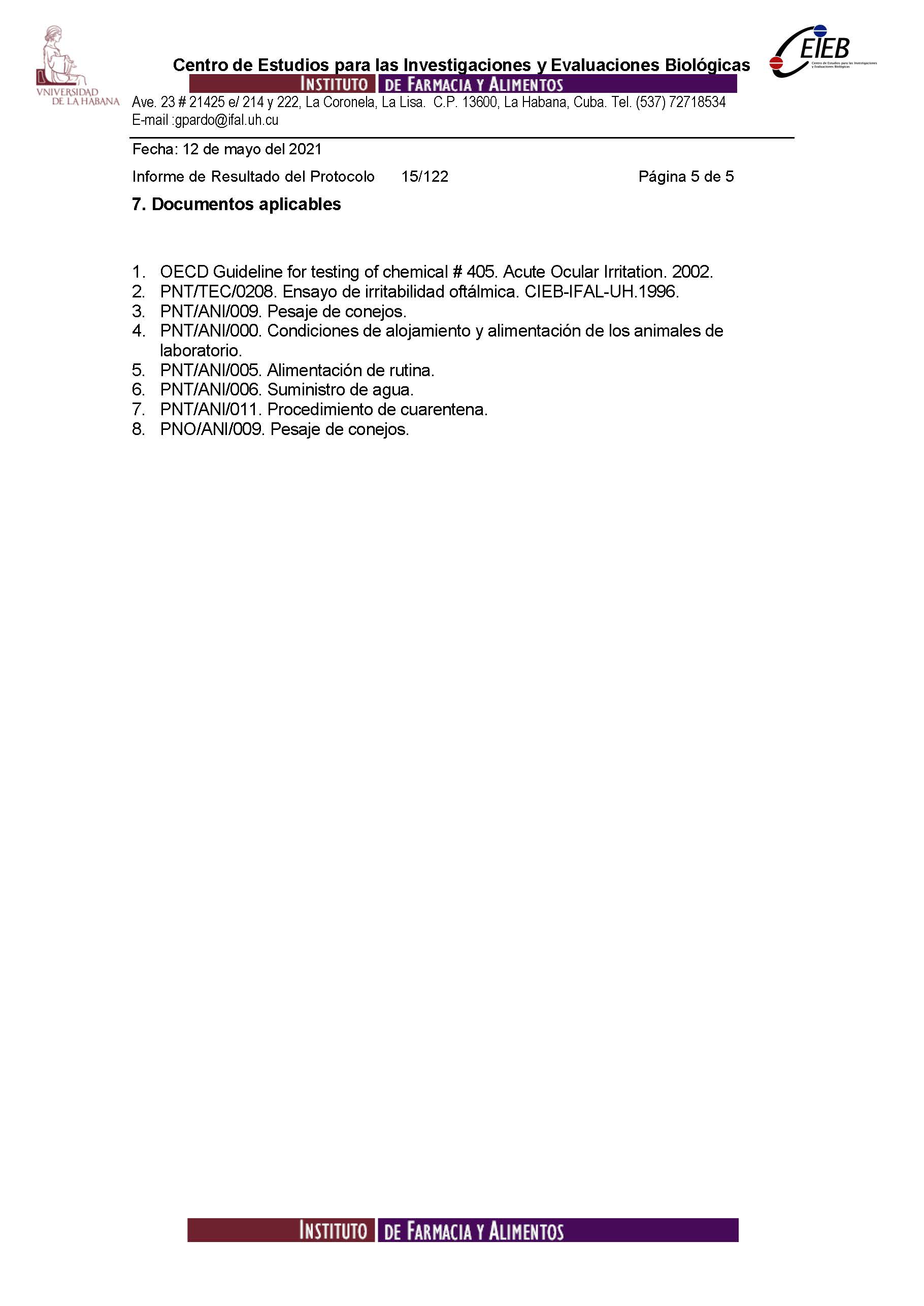

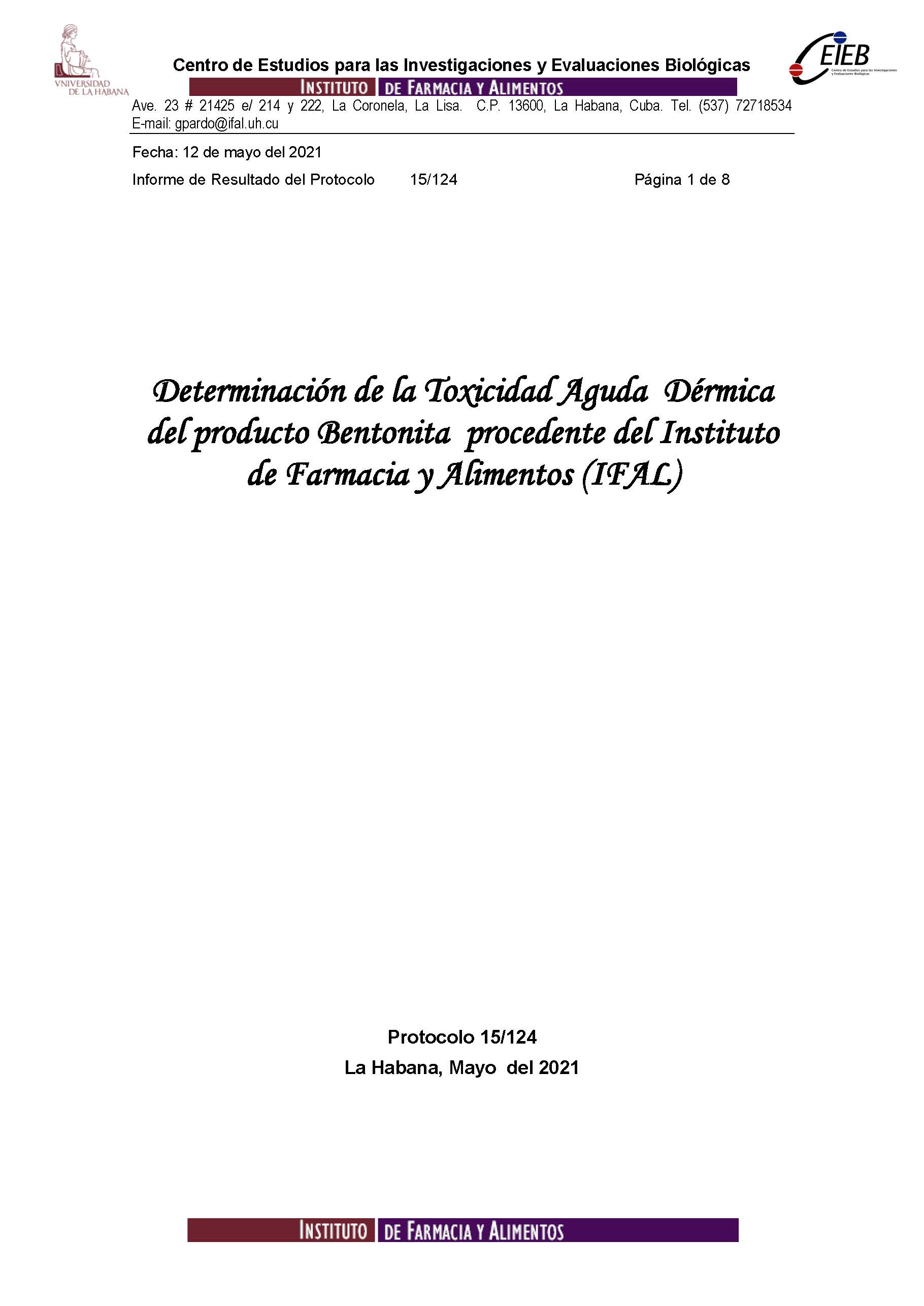
(S3) Acute dermal toxicity


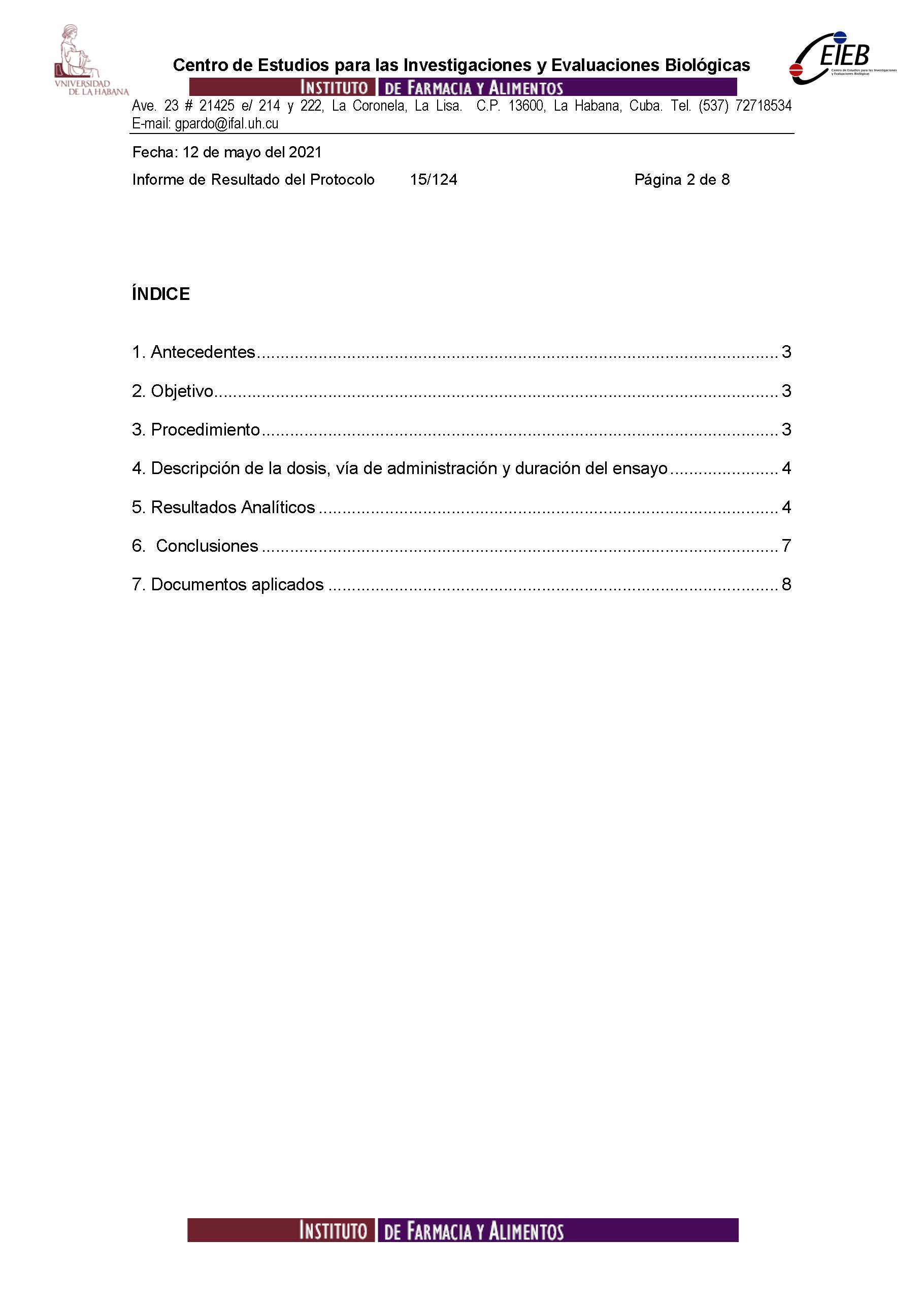

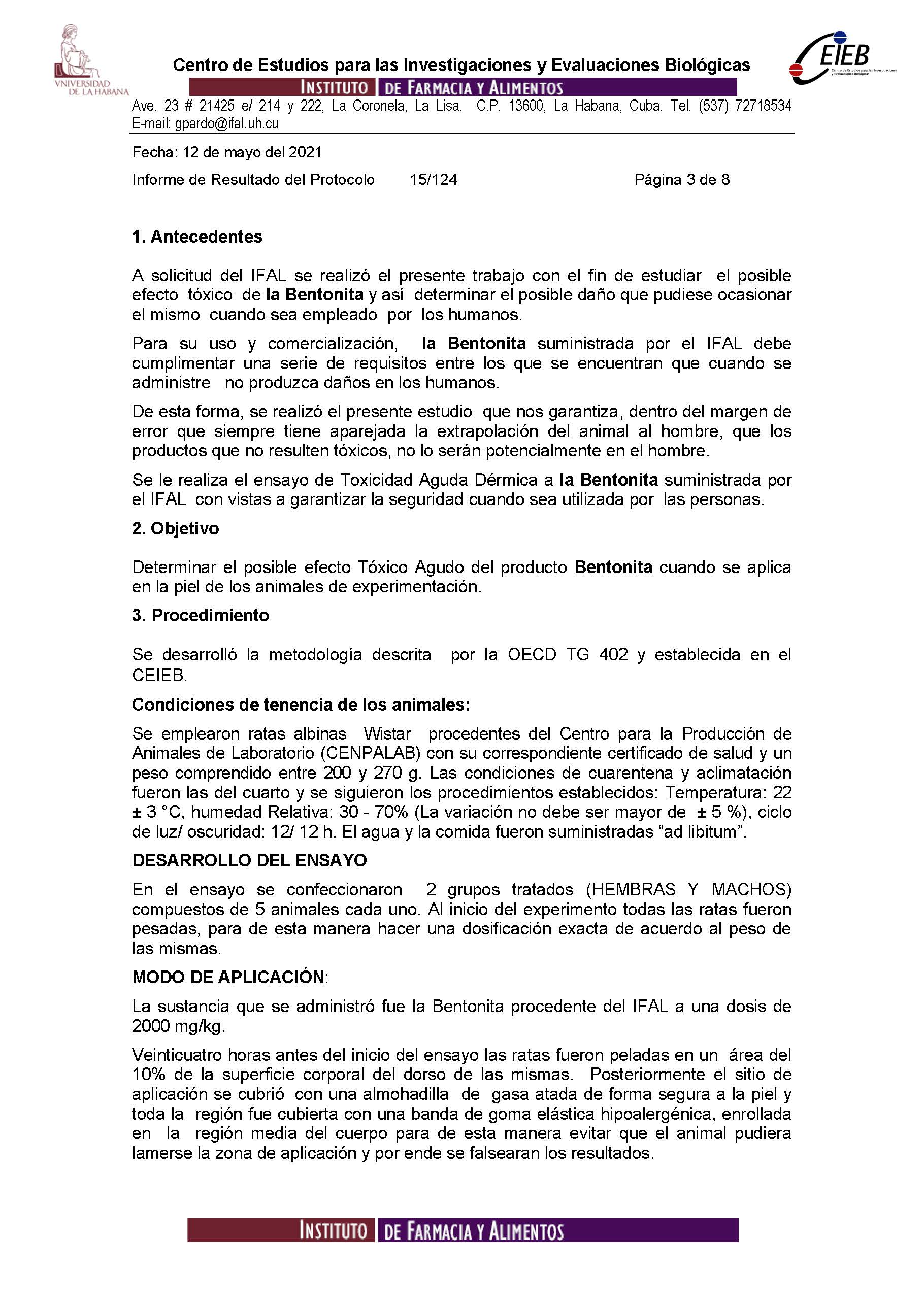

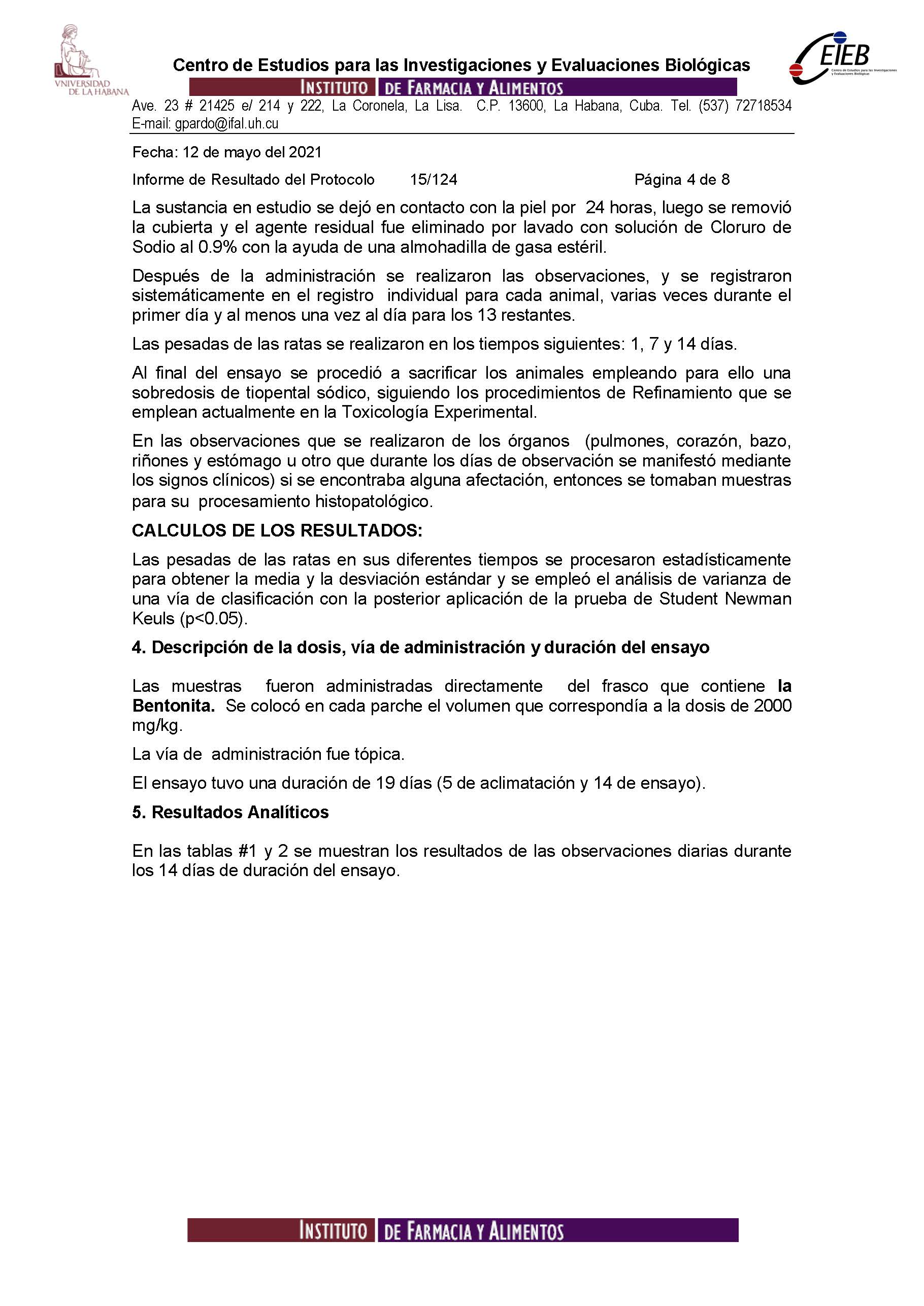

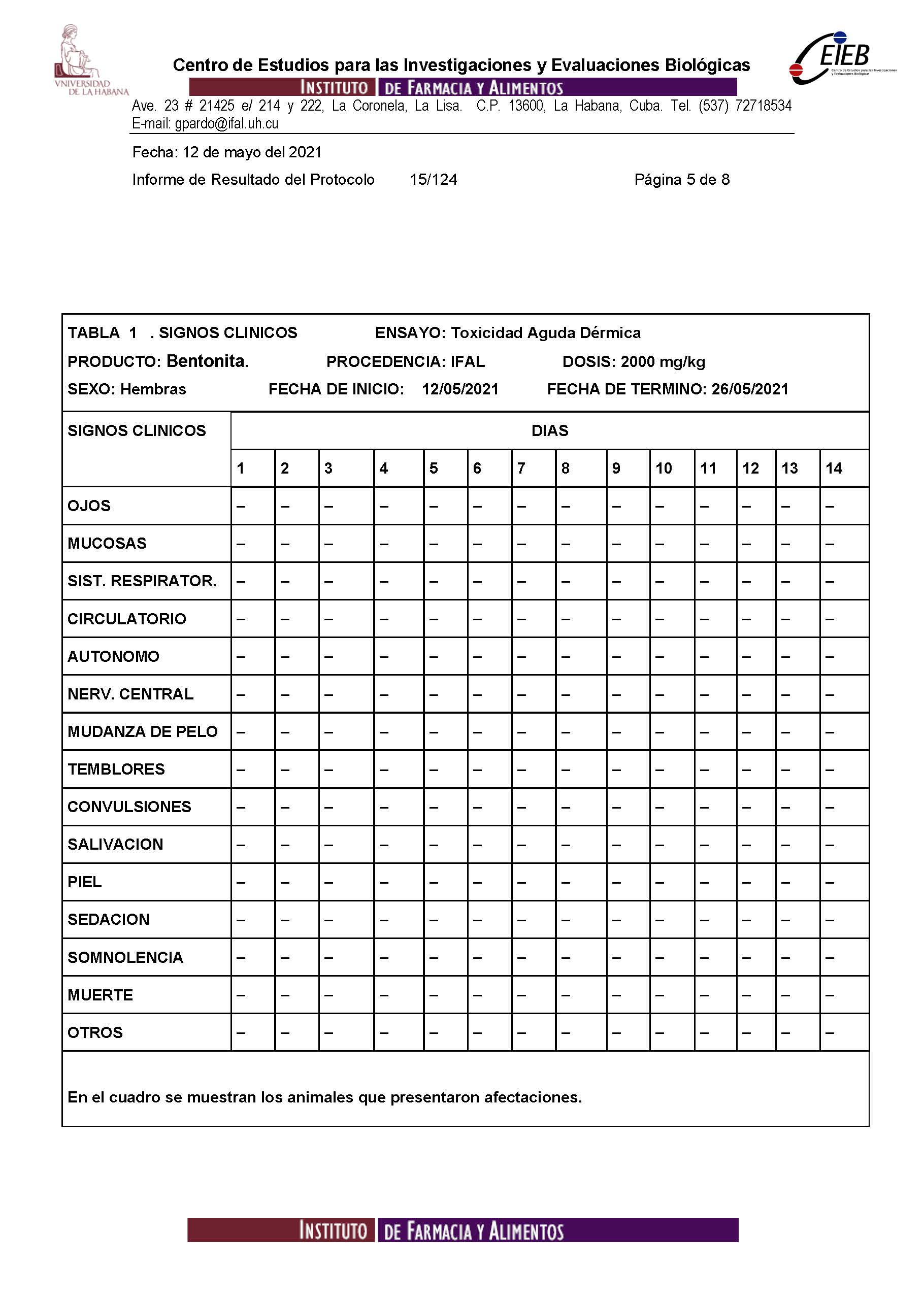

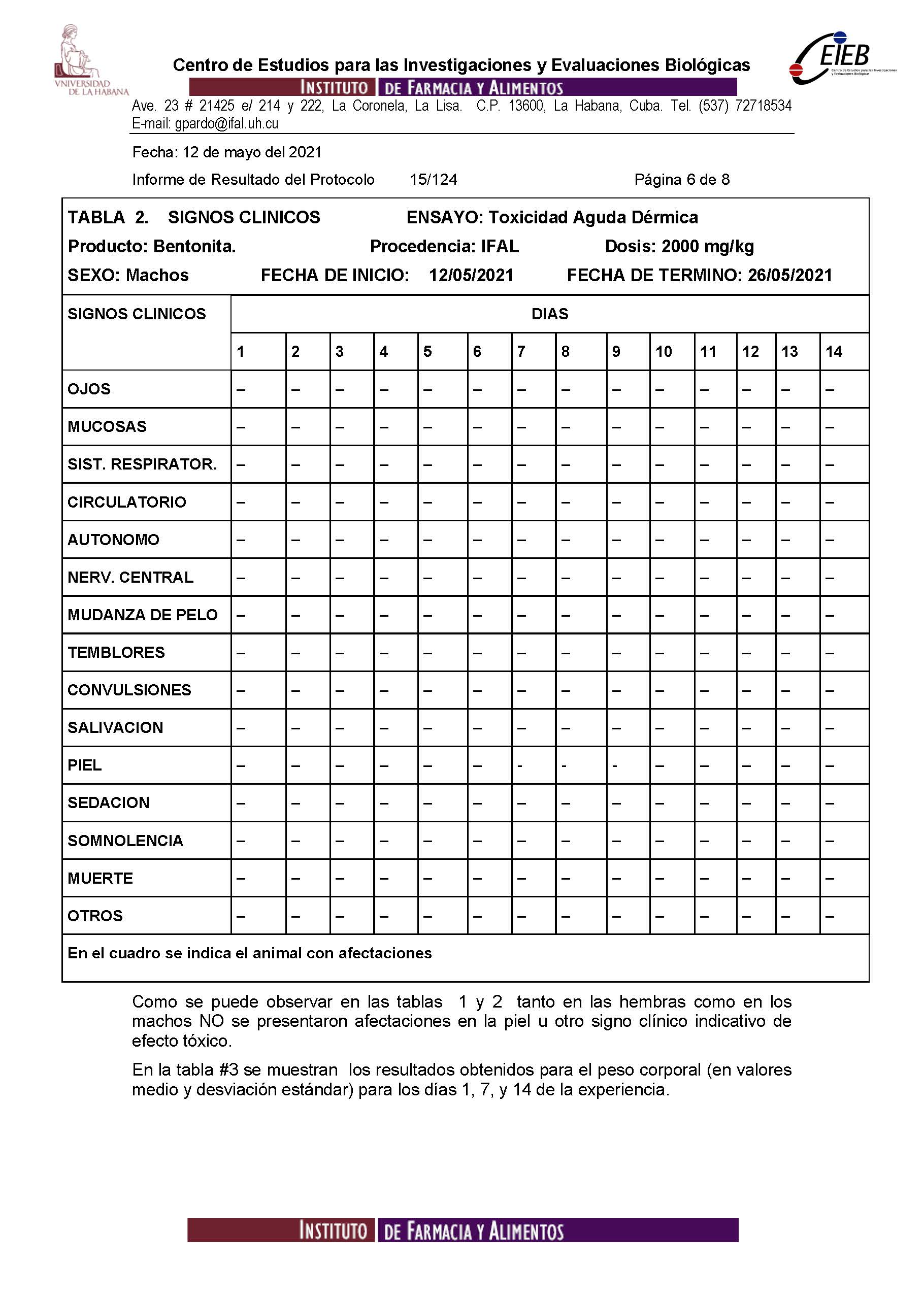

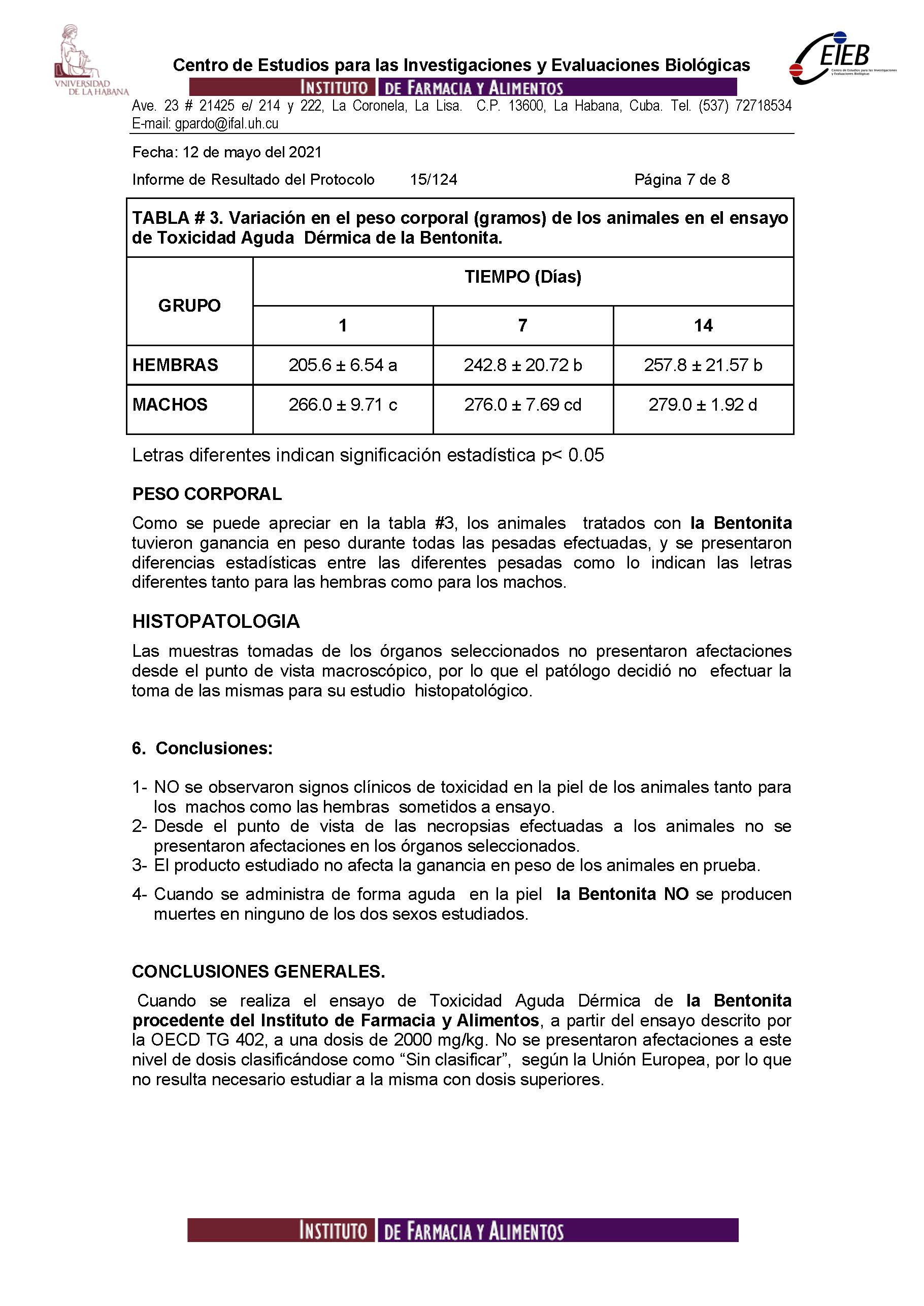

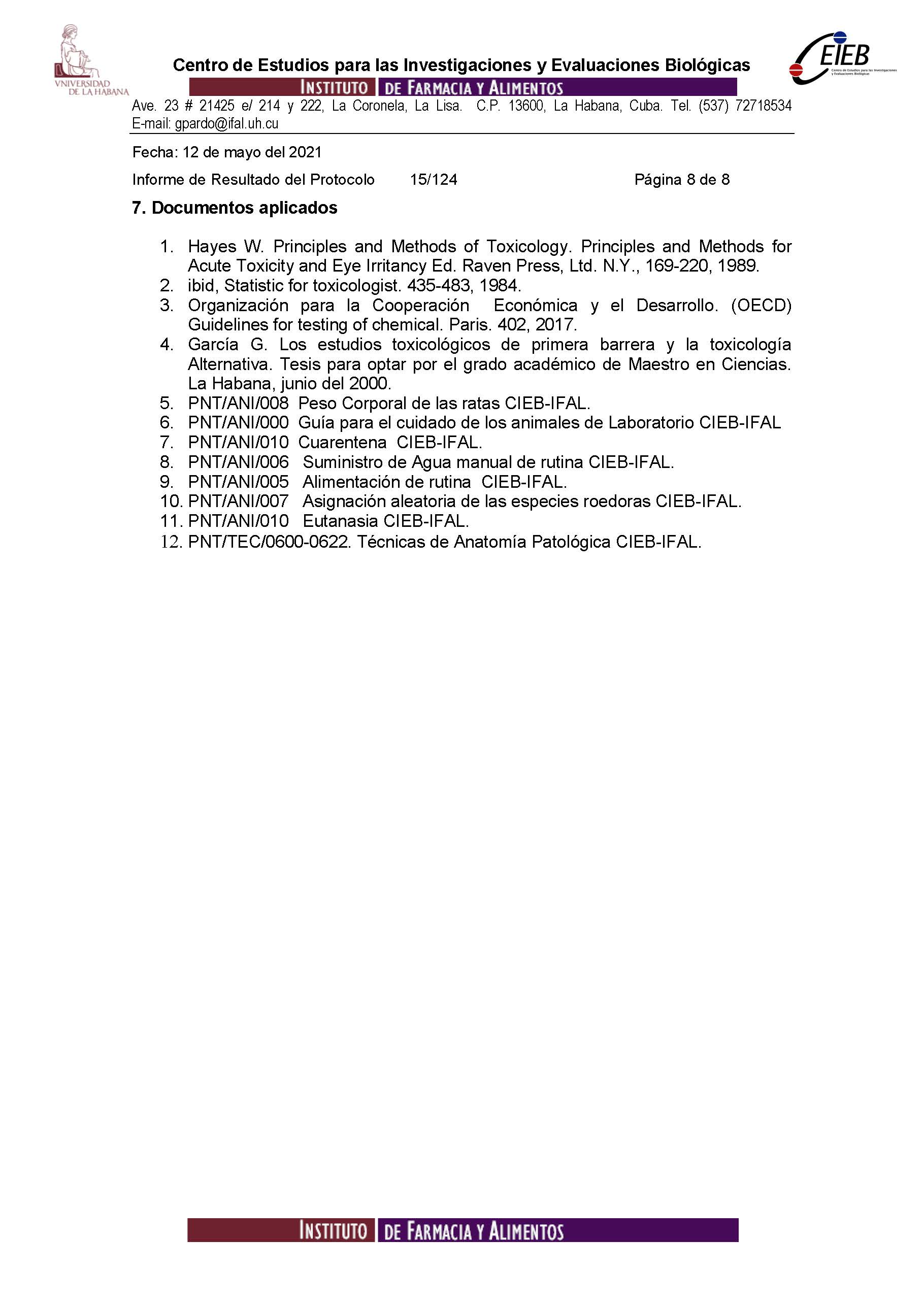

Supplement: Supplementary file 1 [file pharmaceutics-15-01171-s001.zip › pharmaceutics-2212031-supplementary.docx]
